# NeoSSNet: Real-Time Neonatal Chest Sound Separation Using Deep Learning

**DOI:** 10.1109/OJEMB.2024.3401571

**Published:** 2024-05-15

**Authors:** Yang Yi Poh, Ethan Grooby, Kenneth Tan, Lindsay Zhou, Arrabella King, Ashwin Ramanathan, Atul Malhotra, Mehrtash Harandi, Faezeh Marzbanrad

**Affiliations:** Department of Electrical and Computer Systems EngineeringMonash University, Melbourne 2541 Clayton VIC 3800 Australia; Department of Electrical and Computer Systems EngineeringMonash University, Melbourne 2541 Clayton VIC 3800 Australia; BC Children's Hospital Research Institute and the Department of Electrical and Computer EngineeringUniversity of British Columbia 8166 Vancouver BC V6T 1Z4 Canada; Monash Newborn, Monash Children's Hospital and Department of PaediatricsMonash University, Melbourne 2541 Clayton VIC 3800 Australia

**Keywords:** Deep learning, heart sound, lung sound, phonocardiogram (PCG), single-channel sound separation

## Abstract

*Goal:* Auscultation for neonates is a simple and non-invasive method of diagnosing cardiovascular and respiratory disease. However, obtaining high-quality chest sounds containing only heart or lung sounds is non-trivial. Hence, this study introduces a new deep-learning model named NeoSSNet and evaluates its performance in neonatal chest sound separation with previous methods. *Methods:* We propose a masked-based architecture similar to Conv-TasNet. The encoder and decoder consist of 1D convolution and 1D transposed convolution, while the mask generator consists of a convolution and transformer architecture. The input chest sounds were first encoded as a sequence of tokens using 1D convolution. The tokens were then passed to the mask generator to generate two masks, one for heart sounds and one for lung sounds. Each mask is then applied to the input token sequence. Lastly, the tokens are converted back to waveforms using 1D transposed convolution. *Results:* Our proposed model showed superior results compared to the previous methods based on objective distortion measures, ranging from a 2.01 dB improvement to a 5.06 dB improvement. The proposed model is also significantly faster than the previous methods, with at least a 17-time improvement. *Conclusions:* The proposed model could be a suitable preprocessing step for any health monitoring system where only the heart sound or lung sound is desired.

## I. Introduction

Auscultation for neonatal care is critical to physical examinations. It provides access to heart and lung sounds, which can be used to diagnose cardio-respiratory conditions and monitor vital signs. Its application ranges from regular heart-rate assessment [1], [2] to computer-aided diagnosis [3], [4]. These algorithms work best when using high-quality heart or lung sounds, but heart and lung sounds typically only come in pairs and are contaminated by noise. As such, further processing is needed to isolate the individual sound sources.

There are challenges when separating pure heart and lung sounds in newborns: (a) Newborns typically have weak heart and lung sounds due to their smaller organ size. (b) Typical newborn heart sounds have a frequency band between 50 Hz and 250 Hz, while newborn lung sounds have a frequency band between 200 Hz and 1000 Hz [5], causing an overlap in their spectrum. (c) Newborns have a smaller chest area, so focusing the auscultation for the desired heart or lung sound is more difficult. (d) High noise levels in the environment, such as crying noise and respiratory support noise, can interfere with the obtained chest sound mixture.

Traditional chest sound separation methods require heart sound segmentation and lung sound segmentation [5]. For heart sound segmentation, these methods typically identify the first heart sound (S1) and the second heart sound (S2). However, most of these methods struggle in a high-noise scenario. Our recent works showed that using Non-Negative Factorisation (NMF) and Non-Negative Co-Factorisation (NMCF) outperforms these traditional methods when performing chest sound separation in newborn children [6]. Despite that, there are still some limitations. Namely, computation time and the performance in the presence of respiratory support noise remain weaknesses of the method.

Recently, deep learning-based audio source separation has been proposed in various domains. With the success of deep neural networks, they are the state of the art for supervised separation. As a result, domains with large datasets such as those in the speech domain [7], [8], [9] and music domain [10], [11], [12] are dominated by deep neural networks. However, only a smaller amount of data is available for chest sounds from neonatal to adult. If the training data is too small, supervised separation would cause overfitting, thus reducing the model's performance. As such, many different approaches have been proposed to overcome this limitation. For instance, Wang et al. used NMF to aid in the deep learning process [13], while Tsai et al. exploited the periodicity of heart and lung sounds to perform the separation [14]. Adding to this, data augmentation-based learning will be explored in this paper to artificially increase the number of samples and reduce overfitting.

In addition, deep learning-based audio source separation models have been dominated by either convolutional neural networks (CNN) [12], [14] or long short-term memory (LSTM) networks [8], [9]. Typically, CNNs have the advantage of capturing local features well. However, CNNs must be sufficiently deep to capture a desired receptive field. LSTM networks, on the other hand, are capable of learning long-term dependencies. Nonetheless, LSTM networks can suffer from exploding and vanishing gradients, making training difficult. In recent times, state-of-the-art audio encoders have adopted a transformer architecture due to their excellent ability to model sequential data [15], [16]. As such, this paper explores a transformer-based network architecture.

## II. Materials and Methods

### A. Dataset

Raw chest sound recordings were obtained from a previous study by Grooby et al. [6]. 71 chest sounds were collected from newborn babies admitted to Monash Children's Hospital with the approval of the Monash Health Human Research Ethics Committee (HREA/18/MonH/471). The heart and lung sounds were obtained from the recordings via manual annotations and served as the ground truth for training and testing. The Supplementary Material details the collection of the data.

Separately, 33 chest sound recordings containing synchronous vital signs were also collected. The synchronous vital signs collected include second-by-second heart rate from electrocardiogram data and breathing rate from impedance tomography sensors. These chest sounds were further divided into 21 chest sounds without respiratory support sounds and 12 chest sounds with respiratory support sounds.

### B. Model Architecture Overview

**Fig. 1.**
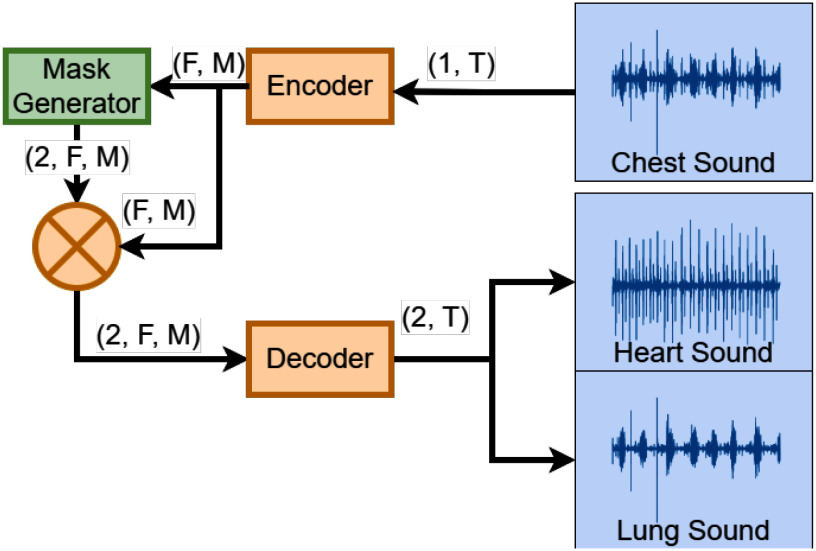
The model architecture used. The model takes in a single-channel input waveform and outputs a heart and lung waveform, separated from the input waveform.

Inspired by the Conv-TasNet model [7], the model architecture is broken down into three components: encoder, decoder, and mask generator. Fig. 1 shows the overall system block diagram. The encoder turns the input waveform of size $(1, T)$ into a 2-dimensional feature space of size $(F, M)$, where $T$ represents the number of samples, $F$ represents the feature dimension, and $M$ represents the number of frames or hops. The mask generator then takes this 2-dimensional feature space and produces 2 feature space masks; one for heart sounds, and one for lung sounds of shape $(2, F, M)$. Each mask is then applied to the feature space and passed to the decoder to transform from the feature space back to the waveform of shape $(2, T)$.

**Fig. 2.**
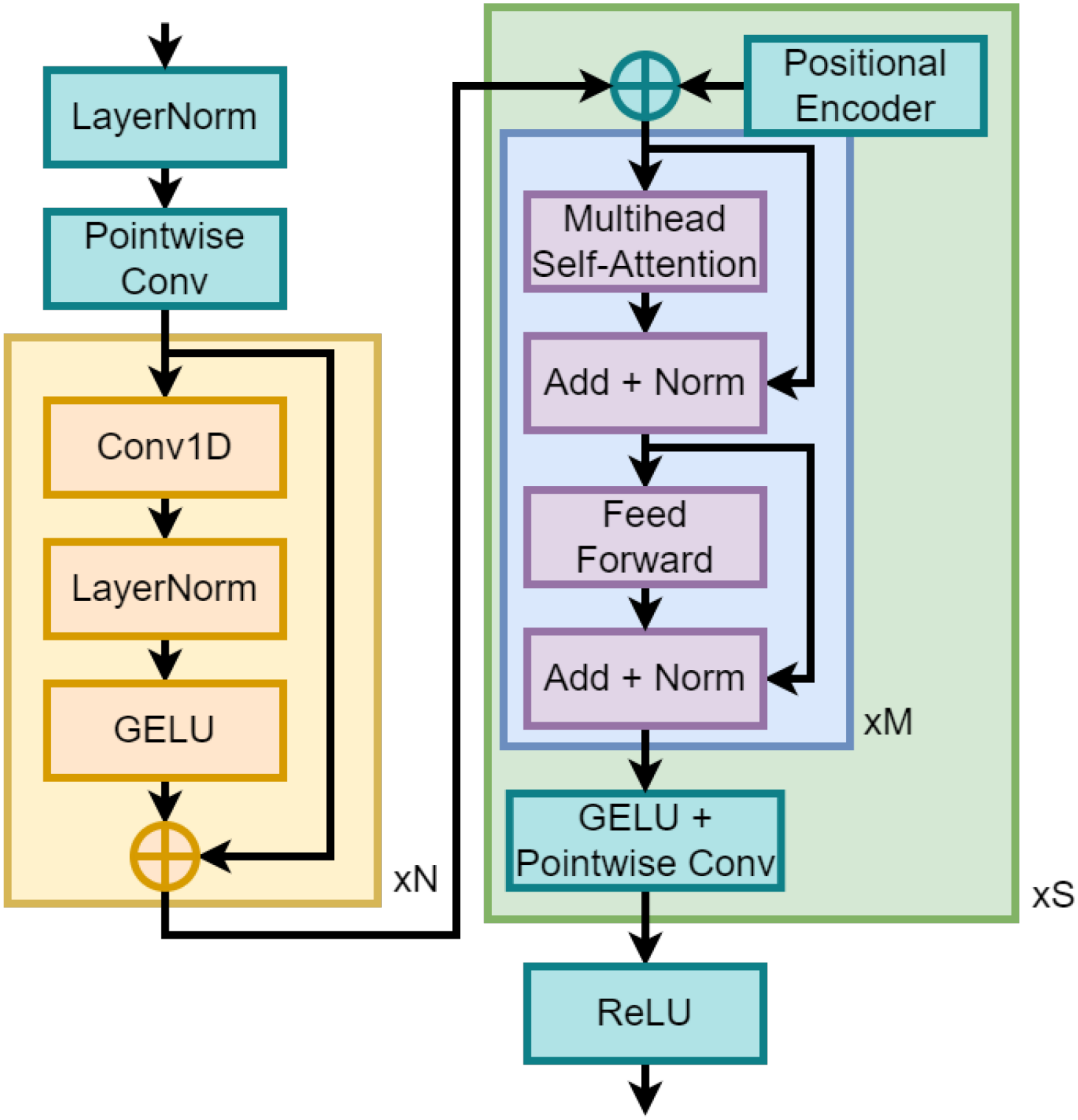
The mask generator in the model. The mask generator takes in the feature space of shape $(F, M)$ and produces $s$ feature space masks of shape $(s, F, M)$, where $s$ is the number of sources. Since we are interested in heart and lung sources, $s=2$.

The encoder and decoder are represented by a 1D convolution and a 1D transposed convolution, while the mask generator architecture is shown in Fig. 2. Further explanation of the model architecture can be found in the Supplementary Material.

### C. Training Configuration

The following modifications were made to the training dataset based on the performance of the trained model on the validation dataset:

1) The reference noise sound in the training dataset was first rescaled to have a relative signal power of −20 dB to 0 dB to ensure that the model was able to learn to identify heart and lung sounds before increasing the relative signal power to be between −10 dB to 10 dB during the fine-tuning phase.
2) Instead of having a discrete relative signal power scaling for lung and noise sound, the signal power scaling was randomly sampled in the specified range.
3) Stethoscope movement noise is removed from the training dataset as it decreases the overall performance of the reconstructed lung sound. Note that the stethoscope movement noise is still present in the test dataset.
4) For the convolutive mixtures, a random filter length was chosen between 3 and 5.
5) Instead of training on the whole 10-second segments, an 8-second segment is randomly cropped and trained on. As such, the model is only trained on samples with a sequence length of 32,000 samples instead of the whole 40,000 samples.

Table I summarises the model parameter selected for the model used. The following hyperparameters were found by sweeping through different combinations of hyperparameters and choosing the best-performing one based on the performance evaluation on the validation dataset.

**TABLE I:** Model Parameter Used for the Final Model

Encoder/Decoder	Value
Kernel Size	512
Feature Size	512
Mask Generator	Value
Mask Feature Size	256
Conv Kernel Size	3
Conv Layers	6
Num Heads	4
Transformer Layers	4

The following training hyperparameters were selected: (a) the model was trained for 40 epochs, (b) The model was trained using the AdamW optimiser with the AMSGrad extension [17] and a weight decay of 0.1, (c) the model was trained with a learning rate scheduler where an initial learning rate of $1 \,\times\, 10^{-4}$ is used, with the learning rate being scaled by 0.5 when the validation accuracy does not improve for 4 epochs, (d) The gradient in the network is clipped if the L2-norm of the gradient is greater than 5, (e) The objective of the training is to maximise the scale-invariant signal-to-distortion ratio (SI-SDR) between the estimated signals $s_{\text{est}}$ and the target signals $s_{\text{target}}$, defined in (1).

\begin{align*}
\alpha &= \frac{s_{\text{est}} \cdot s_{\text{target}}}{\Vert s_{\text{target}}\Vert ^{2}}\\
 e_{\text{noise}} &= \alpha s_{\text{target}} - s_{\text{est}}\\
 \text{SI-SDR} &= 10 \log _{10}{\frac{\Vert \alpha s_{\text{target}}\Vert ^{2}}{\Vert e_{\text{noise}}\Vert ^{2}}} \tag{1}
\end{align*}

### D. Evaluation

The proposed NeoSSNet is compared to the previously proposed NMF and NMCF methods [6]. A short description of the NMF and NMCF methods is included in the Supplementary Material. All separation methods are evaluated in the following categories:

#### 1) Objective Distortion Measures Evaluation

Signal-to-distortion ratio improvement (SDRi) and scale-invariant SDRi (SI-SDRi) were used as objective measures of the performance of the separation method on the artificial data. SI-SDR is defined in (1), while SDR is defined in (2), where the estimated source can be decomposed as shown in (3). Table II contains the description of the decomposed signal.

\begin{align*}
\text{SDR} &= 10 \log _{10}\frac{\Vert s_{\text{target}}\Vert ^{2}}{\Vert e_{\text{interf}} + e_{\text{artif}} + e_{\text{noise}}\Vert ^{2}} \tag{2}
\\
s_{\text{est}} &= s_{\text{target}} + e_{\text{interf}} + e_{\text{noise}} + e_{\text{artif}} \tag{3}
\end{align*}

**TABLE II:** The Description of the Decomposed Estimated Signal

Signal	Description
$s_{\text{est}}$	Estimated sources from the separation algorithm
$s_{\text{target}}$	Target sources with some allowed deformation
$e_{\text{interf}}$	Allowed deformation of sources which accounts for the interference of the unwanted sources
$e_{\text{noise}}$	Allowed deformation of the perturbating noise
$e_{\text{artif}}$	Artifacts of the separation algorithm

The testing partition is further divided into three partitions depending on the type of noise sound present: (1) No Noise: where the input mixture only contains heart and lung sounds. (2) General Noise: where the input mixture contains crying and stethoscope movement noises. (3) Respiratory Support: where the input mixture contains bubble continuous positive airway pressure (CPAP) noise and ventilator CPAP noise.

#### 2) Heart Error Rate and Breathing Error Rate Evaluation

For the 33 real-world data containing vital signs, the heart rate error improvement and breathing rate error improvement were reported as the difference before and after passing through the model compared to the vital signs. The heart rate was estimated using a modified version of the method by Springer et al. [18] suitable for the neonatal heart rate range [19]. The breathing rate was estimated from a 300–450 Hz power spectral envelope every second using peak detection [19].

#### 3) Computation Time Evaluation

For speed comparison, the separation methods were executed on an Intel Core i7-12800H CPU paired with a Nvidia RTX A1000 GPU. For single instances, the input waveform was generated randomly with a length of 40,000 (equivalent to 10 seconds with a sample rate of 4 kHz) and normalised to have values between −1 and 1. For batch instances, the input waveform was processed with a batch size of 16. The batch size then scales down the time taken, and the rescaled time is reported. The overhead of transferring data into memory is omitted for the GPU instance measurement. Every measurement was done ten times, and the mean time taken was reported.

## III. Results

### A. Objective Distortion Measures

**Fig. 3.**
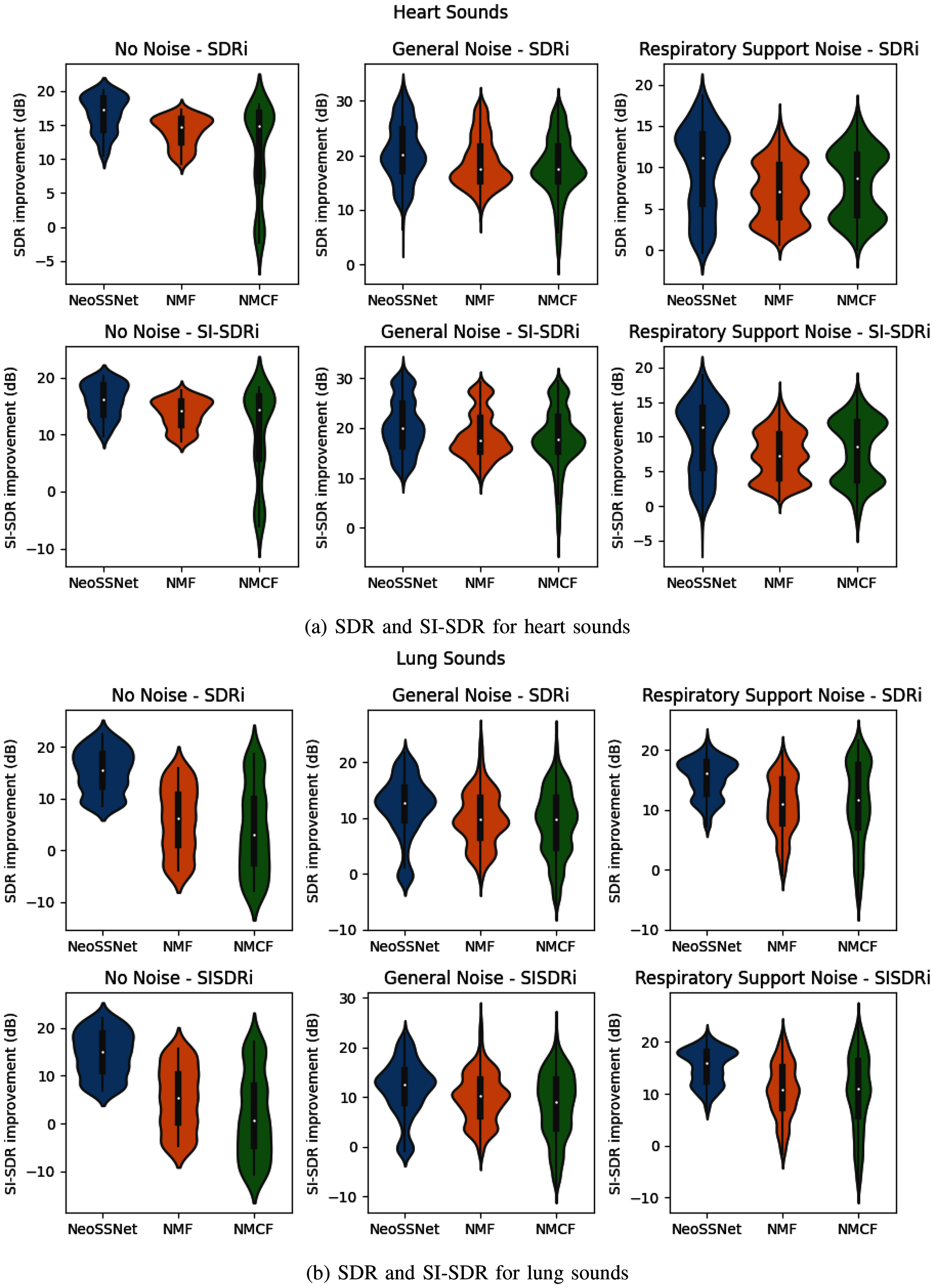
Violin plots of the SDRi and SI-SDRi results for the separated heart and lung sounds. Each violin plot contains the median, interquartile ranges, and the distribution of the SDRi and SI-SDRi results.

We investigate the objective distortion measures of the NeoSSNet with the previous NMF and NMCF methods for neonatal chest sound separation. Fig. 3 show the violin plots for the SDRi and SI-SDRi results for each method in separating heart and lung sounds, while Table III and Table IV shows the median SDRi and SI-SDRi results for the different methods in separating heart and sounds.

**TABLE III:** Median SDRi and SI-SDRi Results for the Heart Sounds Separated From the Artificial Mixture

Noise Type	No Noise			General Noise			Respiratory Support Noise		
Methods	NeoSSNet	NMF	NMCF	NeoSSNet	NMF	NMCF	NeoSSNet	NMF	NMCF
SDR (dB)	**17.24**	14.64	14.85	**20.16**	17.42	17.57	**11.77**	7.04	8.67
SI-SDR (dB)	**16.21**	14.23	14.36	**19.81**	17.60	17.80	**11.99**	7.30	8.53

**TABLE IV:** Median SDRi and SI-SDR Results for the Lung Sounds Separated From the Artificial Mixture

Noise Type	No Noise			General Noise			Respiratory Support Noise		
Methods	NeoSSNet	NMF	NMCF	NeoSSNet	NMF	NMCF	NeoSSNet	NMF	NMCF
SDR (dB)	**15.50**	6.14	3.07	**12.71**	9.81	9.72	**16.08**	11.02	11.68
SI-SDR (dB)	**15.01**	5.40	0.74	**12.47**	10.19	9.12	**15.90**	10.79	10.93

The NeoSSNet model outperforms previous methods in the objective distortion measure across all aspects. In particular, the NeoSSNet performed better in the presence of respiratory support noise and in separating lung sounds without noise.

### B. Heart Rate and Breathing Rate Analysis

We study the effect of applying model separation algorithms to improve the accuracy of the heart-rate estimation algorithm and breathing-rate estimation algorithm. Table V shows the heart rate improvement (HRi) and breathing rate improvement (BRi) for the real-world chest sounds for each separation method when compared to the vital signs collected.

**TABLE V:** The Mean Heart Rate and Breathing Rate Improvement in Beats Per Minute (bpm).

Noise Type	Nil (bpm)		Resp (bpm)	
Metric	HRi	BRi	HRi	BRi
NeoSSNet	**2.62**	**1.89**	3.29	**0.10**
NMF	1.92	1.45	2.65	-1.04
NMCF	2.24	1.27	**5.47**	-1.30

Nil Signified That No Respiratory Support Machines Were Present During the Collection of the Chest Sounds. Resp Signified That Respiratory Support Machines Were Present During the Collection of the Chest Sounds

For heart rate improvements, when there is no respiratory support noise, the NeoSSNet performed better, while the previous NMCF method performed better with respiratory support. However, the NeoSSNet model performs better regarding breathing rate improvement than the previous methods in both scenarios.

### C. Computation Time

We analyse the computation times for different methods. Table VI shows the computational time of the proposed method compared to the previous methods.

**TABLE VI:** Computation Time Comparisons.

Method	NeoSSNet	NMF	NMCF
Single Instance	42.04 ms	714.0 ms	23.92 s
Batch Instance	18.42 ms	N/A	N/A
Single Instance (GPU)	23.88 ms	N/A	N/A
Batch Instance (GPU)	1.320 ms	N/A	N/A

All Measurements Were Made in Milliseconds Except for the NMCF Method, Which is Made in Seconds

The NeoSSNet model is significantly faster than the previous methods. For the single instance case, the proposed model is 17 times faster than the NMF method and 570 times faster than the NMCF method. Additionally, the proposed model benefits from batch processing and GPU support, further increasing the computation speed compared to the previous methods.

### D. Model Training and Optimisation

We study the effects of different model parameters and training configurations on the model's performance. Table VII shows the objective distortion measurements for the different modifications made to the baseline model described in Table I. From the table, we observe the following:

**TABLE VII:** The Effect of Different Model Configurations.

Changes	Properties	Metric			
	Model Size	SDR Heart	SDR Lung	SI-SDR Heart	SI-SDR Lung
	(M)	(dB)	(dB)	(dB)	(dB)
Baseline	8.42	**16.39**	14.76	**16.00**	14.46
Removed conv in mask generator, added transformer layers to compensate for smaller model size	10.40	14.25	14.32	13.91	13.96
Changed encoder/decoder to short-time Fourier transform/ inverse short-time Fourier transform (STFT/iSTFT)	7.90	13.31	14.39	13.01	14.20
Decrease encoder kernel size to 256	8.16	15.61	**15.04**	15.39	**14.57**
Increase encoder kernel size to 1024	8.95	16.05	12.55	15.51	11.63
Decrease feature space size to 256	7.96	15.95	14.75	15.52	14.08
Increase feature space size to 1024	9.34	15.80	14.37	15.47	14.05
Trained with relative SNR noise from -10 dB to 10 dB	8.42	15.97	13.35	15.67	12.57

The Changes Were Made From the Baseline Model Configuration Described in Table I

1) The convolution model before the transformer is important to improve the model's performance.
2) The use of convolution/transposed convolution for the encoder/decoder pair improves the model's performance, especially in the respiratory support noise cases, where the performance of the model. In particular, the following is the breakdown of the result (SDR-Heart: 5.66 dB, SDR-Lung: 13.21 dB, SI-SDR-Heart: 5.86 dB, SI-SDR-Lung: 13.46 dB).
3) A smaller kernel size has a small improvement to the performance of the lung sound separation.
4) An optimal model performance is achieved with a feature size of 512.

## IV. Discussion

Our findings demonstrated improvements based on objective measures of the separation method compared to previous methods. In particular, we addressed our previous limitations in handling respiratory support noises. We theorised that this improvement comes from the use of convolution-based encoder/decoder architecture rather than the traditional STFT/iSTFT-based encoder/decoder architecture. This is further supported in the model parameter section, where changing the encoder/decoder back to STFT/iSTFT causes the model to regress to the performance of previous methods. Therefore, we hypothesised that STFT/iSTFT is not optimal when performing chest sound separation in the presence of respiratory support noise. Instead, the linear transformations learned by convolution-based encoder/decoder are optimal.

The findings did not meet expectations when observing the heart rate and breathing rate algorithm analysis. Despite observing some performance enhancement, the performance improvement falls short of expectations. This result could be due to a few factors: (a) In the heart rate improvement case, the heart rate estimation algorithm is already robust to noises, and all three algorithms only work to improve the outlier samples. (b) The chest sound quality for most samples was low, with minimal to nonexistent detection of both heart and lung sounds. This is especially true for the respiratory support samples, where the respiratory support machine noises dominated the chest sound recordings. Notwithstanding the foregoing, these samples are typically what is expected, and further improvement has to be made here to improve the performance of these models. This highlights that higher objective measures do not directly correlate with enhancing the performance of algorithms, and the performance of these algorithms can be down to many factors.

One definitive metric that the proposed model handily outperforms previous methods is in computation time. This is because NMF and NMCF require gradient descent to perform matrix factorisation. This significantly increases the computational requirements of NMF and NMCF, and as such, NeoSSNet can perform much faster.

**Fig. 4.**
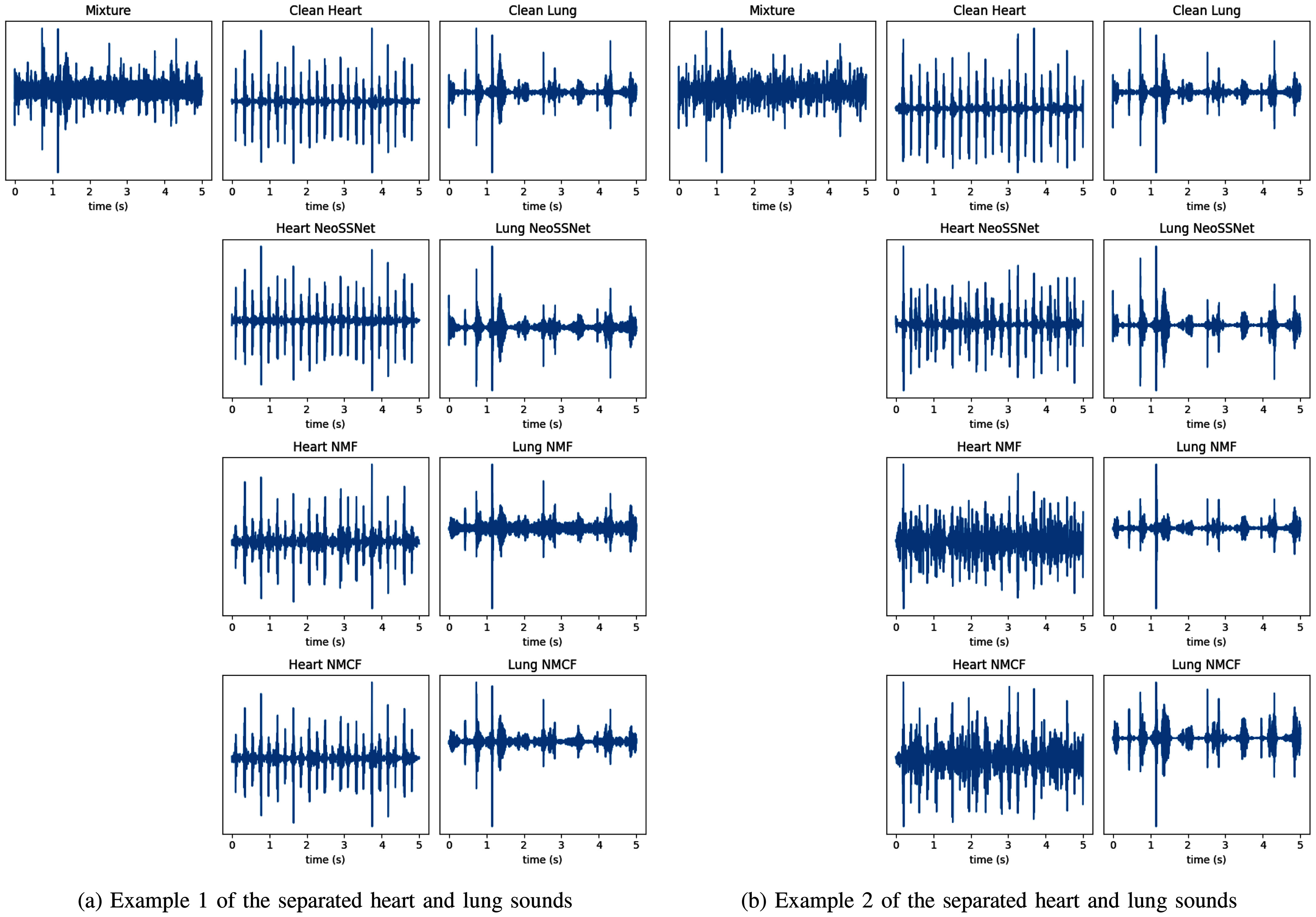
A comparison of the separated heart and lung sounds in the presence of the respiratory support noise.

Fig. 4 shows two separated chest sounds in the presence of respiratory support noise. Overall, the heart and lung sounds generated by NeoSSNet are cleaner, with less background noise, compared to the previous methods. Fig. 4(b) showcases where the heart sounds generated by NeoSSNet outperform the previous methods, where the separated heart sounds still contain a significant amount of noise. As such, we observed significant improvements in the separation performance of NeoSSNet when compared to previous methods in the presence of respiratory support noise.

### A. Future Works

Although the model performed well with artificial chest sound mixtures, there is still room for improvement in its real-world chest sound performance. A simple idea here is to incorporate real-world metrics such as heart error rate, breathing error rate, or subjective signal-quality metrics into the loss function to improve the model's performance for real-world use.

One limitation of the NeoSSNet when generating heart sounds is the possible insertion of phantom heartbeats. This can be seen in Fig. 4(b), where extra S2 beats are inserted into the separated heart sounds. As such, more physics-informed learning will be explored in the future to ensure that the separated chest sounds will follow our current understanding of heart and lung sounds.

Lastly, we acknowledge that our current models may exhibit biases due to being trained on a particular set of demographics using only a single type of digital stethoscope. The dataset only consists of newborn babies admitted to Monash Children's Hospital and recorded using a CliniCloud stethoscope. As such, the model's current applicability and accuracy are limited. In future work, we aim to use more diverse open-source phonocardiogram datasets from different parts of the world using various digital stethoscope brands to expand the diversity of the dataset and reduce the model bias.

## V. Conclusion

We conclude that the proposed deep learning-based sound separation method represents an advancement in neonatal chest sound separation compared to previous methods. These improvements suggest that the proposed model could replace previous neonatal chest sound separation methods. For example, our model's improved objective distortion measurements imply that the separated heart and lung sounds are of better quality than previous attempts, potentially making them suitable as a preprocessing step for various algorithms involving phonocardiogram-based health monitoring systems. Additionally, the significantly lower computational costs suggest that the proposed model could be ideal for real-time applications. Nevertheless, subjective signal-quality measurements and exploring a physics-informed neural network remain uncharted territory, which may help bridge the gap between real-world chest sound separation and the removal of noisy ground truth samples.

## Supplementary Materials

The supplementary material contains the following items: (1) some basic background on the NMF and NMCF methods, (2) the data collection process, and (3) further details on the model architecture.

## Conflict of Interest

All authors declare that they have no conflict of interest.

## Author Constributions

Y. P. developed and implemented the NeoSSNet model, conducted the experiments, and wrote the first draft of the manuscript. E. G. contributed to pre-processing and conducted experiments/comparisons. A. M. designed the clinical study, provided clinical insights, and contributed to the manuscript revision. K. T., L. Z., A. K., and A. R. contributed to the clinical experiments and data collection. M. H. and F. M. provided guidance for model development and enhancement, as well as revision of the manuscript. All authors reviewed and approved the manuscript.
